# Is there a Role for Antenatal Corticosteroids in Term Infants before Elective Cesarean Section?

**DOI:** 10.1055/s-0041-1726055

**Published:** 2021-05-12

**Authors:** Augusta Arruda, Mariana Ormonde, Sarah Stokreef, Beatriz Fraga, Catarina Franco, Catarina Dâmaso, Ana Lima

**Affiliations:** 1 Pediatrics Department, Hospital Divino Espírito Santo, Ponta Delgada, Portugal; 2 Obstetrics & Gynecology Department, Hospital Divino Espírito Santo, Ponta Delgada, Portugal

**Keywords:** elective cesarean section delivery, neonatal outcomes, antenatal corticosteroids

## Abstract

**Objective** Cesarean section (CS) delivery, especially without previous labor, is associated with worse neonatal respiratory outcomes. Some studies comparing neonatal outcomes between term infants exposed and not exposed to antenatal corticosteroids (ACS) before elective CS revealed that ACS appears to decrease the risk of respiratory distress syndrome (RDS), transient tachypnea of the neonate (TTN), admission to the neonatal intensive care unit (NICU), and the length of stay in the NICU.

**Methods** The present retrospective cohort study aimed to compare neonatal outcomes in infants born trough term elective CS exposed and not exposed to ACS. Outcomes included neonatal morbidity at birth, neonatal respiratory morbidity, and general neonatal morbidity. Maternal demographic characteristics and obstetric data were analyzed as possible confounders.

**Results** A total of 334 newborns met the inclusion criteria. One third of the population study ( *n* = 129; 38.6%) received ACS. The present study found that the likelihood for RDS (odds ratio [OR] = 1.250; 95% confidence interval [CI]: 0.454–3.442), transient TTN (OR = 1.,623; 95%CI: 0.556–4.739), and NIUC admission (OR = 2.155; 95%CI: 0.474–9.788) was higher in the ACS exposed group, although with no statistical significance. When adjusting for gestational age and arterial hypertension, the likelihood for RDS (OR = 0,732; 95%CI: 0.240–2.232), TTN (OR = 0.959; 95%CI: 0.297–3.091), and NIUC admission (OR = 0,852; 95%CI: 0.161–4.520) become lower in the ACS exposed group.

**Conclusion** Our findings highlight the known association between CS-related respiratory morbidity and gestational age, supporting recent guidelines that advocate postponing elective CSs until 39 weeks of gestational age.

## Introduction

Cesarean section (CS) delivery, especially without previous labor, is associated with worse neonatal respiratory outcomes. 1 2 3 4 5 6 7 In fact, the World Health Organization (WHO) statement on CS rates concluded that CS rates > 10% are not associated with reductions in maternal and newborn mortality rates and may be harmful. 8 Nonetheless, the CS rates have been increasing in the last decades. 9

There is evidence that the risk for CS-related neonatal morbidity is associated with gestational age, with higher risks in lower gestational ages. 10 11 As a result, the majority of guidelines recommend performing elective CS at or after 39 + 0 weeks of gestation to reduce respiratory morbidity. 12 13 14

There is some evidence that, during labor, neurohormonal mechanisms involving the activation of sodium channels are responsible for alveolar fluid clearence. 15 16 Corticosteroids appear to increase the number and function of these sodium channels, as well as their response to catecholamines and thyroid hormones. 16 17 This is considered to be one of the reasons behind the potential utility of antenatal corticosteroids (ACS) in elective CS.

Studies comparing neonatal outcomes between term newborns exposed and not exposed to ACS before elective CS are sparse. Four trials (3,956 women and 3,893 neonates) comparing prophylactic administration of betamethasone or dexamethasone versus placebo with usual treatment without steroids in term elective CS revealed that prophylactic ACS administration appeared to decrease the risk of respiratory distress syndrome (RDS), transient tachypnea of the newborn (TTN), admission to the neonatal intensive care unit (NICU), and the length of stay in the NICU. 18 19 20 21 22 23 Nonetheless, a follow-up study to the ASTECS trial found that the use of ACS before elective CS was associated with lower school performance. 24

Despite the promising findings in the aforementioned clinical trials, there is not sufficient evidence yet, regarding effectiveness or safety, to support the use of ACS in term elective CS. Unfortunately, the available guidelines for clinical practice are not coherent regarding this subject. 12 13 14

## Methods

We performed an observational retrospective cohort study.

We selected all neonates delivered through elective CS between 37 0/7 and 38/7 weeks of gestation at the Hospital Divino Espírito Santo of the Ponta Delgada EPER between January 1, 2012, and December 31, 2017. All elective CSs were included regardless of the indication for elective CS or presence of maternal comorbidity/pregnancy complications. The exclusion criteria were: unknown pregnancy until time of birth, pregnancy with known congenital or chromosomic disorders, multifetal pregnancy, and mothers on systemic corticotherapy for reasons other than pulmonary maturation of the fetus. The charts of both mothers and newborns who met the inclusion criteria were reviewed by one of the three investigators.

The present study was approved by the Hospital Divino Espírito Santo of the Ponta Delgada EPER ethics committee for health.

We divided our population of selected newborns into two: (a) those exposed to ACS for pulmonary maturation; and (b) those not exposed. The decision of using ACS for lung maturation was made individually by the obstetric team.

For primary outcomes, we evaluated the morbidity of the newborns at birth and the respiratory morbidity in the first 72 hours. Variables evaluating morbidity of the newborns at birth included: Apgar score (AS) at the 1 ^st^ and 5 ^th^ minutes of life, need for oxygen supplementation, need for intermittent positive airway pressure (IPPV), and endotracheal intubation. Respiratory morbidity in the first 72 hours of life included: need for ongoing respiratory support (including oxygen, invasive or noninvasive respiratory support), surfactant administration, and occurrence of RDS and TTN (as registered in the charts of the newborns).

Regarding secondary outcomes, we evaluated the occurrence of the following outcomes during hospital stay: need of a prolonged staying in the hospital (in days); need for admission at the NIUC; occurrence of hypoglycemia (documented glucose < 45 mg/dL); sepsis evaluation (screening complete blood count, blood culture, or both); treatment with antibiotics for presumed sepsis; nutrition through nasogastric tube; and treatment for hyperbilirubinemia with phototherapy.

Due to the expected low frequencies of the different outcomes in the study population, we also combined the various outcomes into three composite adverse outcomes. The composite morbidity at birth outcome combines: the need for oxygen supplementation with need for IPPV, need for EOT, and AS < 7 at the 1 ^st^ and 5 ^th^ minutes of life. The composite respiratory morbidity outcome combines: the need of oxygen supplementation with need for noninvasive respiratory support; need for invasive respiratory support; need for surfactant therapy; and occurrence of RDS and/or TTN in the newborn. Finally, the composite general neonatal morbidity outcome combines: prolonged hospital stay (in days) with admission at the NIUC; nutrition through nasogastric tube; jaundice with need for phototherapy; sepsis evaluation; treatment with antibiotics; and hypoglycemia.

We also collected data regarding the drug use for pulmonary maturation (dexamethasone or betamethasone), the number of dosages taken by the mother – one or two for betamethasone and one, two, three or four for dexamethasone – and gestational age at the time of completion of the ACS cycle.

Maternal demographic characteristics and obstetric data were also collected and analyzed as possible confounders, namely: age and race of the mother; prior maternal history of premature delivery; prior maternal history of cesarean delivery; maternal hypertensive disease (chronic, gestational or preeclampsia); maternal diabetes (pre-existing or gestational); premature rupture of membranes; maternal history of hemorrhage during the first, second or third trimester; oligohydramnios; fetal growth restriction; and short cervix. Other data analyzed as possible confounders were birth weight and gender of the newborns.

Data was analyzed using IBM SPSS Statistics for Windows, version 23.0 (IBM Corp., Armonk, NY, USA). Differences between groups for categorical variables were tested using either the χ2 or the Fisher exact tests. Multivariable logistic regression was also performed to estimate the odds for RDS, TTN, NIUC admission, prolonged hospital stay (≥ 5 extra days), and the composite morbidity outcomes in the ACS-exposed and not exposed newborns, adjusting for confounders with statistical differences between the two groups.

## Results

For the study period, 334 newborns met the inclusion criteria. More than one third of the population study ( *n* = 129; 38.6%) received ACS for pulmonary maturation. For these, the drug of choice was betamethasone in 81% ( *n* = 94). The ACS cycle was completed in 93.7% ( *n* = 104) of the exposed neonates. For those who completed the ACS cycle, the number of days between ACS and birth was < 2 days in 28.8% ( *n* = 30), between 2 and 7 days in only 37.5% ( *n* = 39), and > 7 days in 33.6% ( *n* = 35). The main reason for elective CS in the present study ( Table 1 ) was previous uterine surgery (41.3%), namely previous CS, followed by fetal malpresentation and malposition (25.5%).

**Table 1:** Major indication for elective cesarian section

Major indication for elective cesarian section ( *n* = 334)	
Previous uterine surgery	138 (41.3%)
Fetal malpresentation and malposition	85 (25.5%)
Unspecified	63 (18.8%)
Maternal contraindication for vaginal delivery	28 (8.4%)
Pregnancy associated comorbidity	12 (3.6%)
Suspected fetal macrosomia	8 (2.4%)

The majority of the study population (79.3%) had a gestational age ≥ 38 weeks ( Table 2 ). However, there was a statistically significant difference between the groups (p-value [ *p* ] = < 0.001; 95%CI), with the ACS-exposed group having more neonates born with gestational age < 38 weeks (32.1% versus 12.9%, respectively). Regarding the birthweight, only 6.5% of all newborns had low birthweight, with no statistical differences between groups ( Table 2 ).

**Table 2:** Perinatal data of all the newborns included in the study and comparison between the two interest groups

Perinatal data				
	All ( *n * = 334)	Group with antenatal corticosteroids ( *n* = 129)	Group without antenatal corticosteroids ( *n* = 205)	*p-value* *
Gestational age				< 0.001
(weeks)				
37 0/7–37 6/7	69 (20.7%)	42 (32.1%)	27 (12.9%)	
38 0/7–38 6/7	265 (79.3%)	87 (67.9%)	178 (87.0%)	
Birthweight (grams)				0.12
≥2500	312 (93.5%)	117 (90.7%)	195 (95.2%)	
< 2500	22 (6.5%)	12 (9.3%)	10 (4.9%)	
Gender	F 185 (55.4%) M 149 (44.6%)	F 78 (60.5%) M 51 (39.5%)	F 107 (52.2%) M 98 (47.8%)	0.120

Abbreviations: F, female; M, male.

* P-value represents χ2 or Fisher test statistics of comparison among two groups for categorical variables

There were no differences between groups for most of the variables regarding maternal demographic characteristics, with the exception of short cervix ( *p* = 0,014; 95%CI), threatened preterm birth ( *p* =  < 0.001, 95% CI) and arterial hypertension ( *p* = 0.005, 95%CI ( Table 3 ).

**Table 3:** Maternal demographic and obstetric data of the mothers of all newborns included in the study and comparison between the two groups of interest

*Maternal demographic and obstetric data*				
Variables	All ( *n* = 334)	Group with ACS ( *n* = 129)	Group without ACS ( *n* = 205)	*p-value* *
Age (years old) > 34 20–34 ≤ 20	129 (38.6%) 206 (61.7%) 9 (2.7%)	59 (45.7%) 68 (52.7%) 2 (1.6%)	70 (34.1%) 138 (67.3%) 7 (3.4%)	0.08
Ethnicity Caucasian African American Others	305 (98.4%) 4 (1.2%) 1 (0.3%)	121 (99.2%) 1 (0.8%) 0 (0%)	190 (98%) 3 (1.5%) 1 (0.5%)	0.604
Short cervix Yes No	9 (2.7%) 325 (97.2%)	7 (5.4%) 122 (94.6%)	2 (1%) 203 (99%)	0.014
Threatened preterm birth Yes No	19 (5.6%) 315 (94.4%)	15 (11.6%) 114 (88.4%)	4 (2%) 201 (98%)	<0.001
Premature rupture of membranes Yes No	2 (0.6%) 132 (99.4%)	0 (0%) 129 (100%)	2 (1%) 203 (99%)	0.524
Uterine bleeding during pregnancy Yes No	18 (5.4%) 340 (94.6%)	8 (6.2%) 122 (94.6%)	10 (4.9%) 11 (95.1%)	0.602
Oligohydramnios Yes No	7 (2.1%) 327 (97.9%)	3 (2.1%) 126 (97.9%)	4 (2%) 201 (98%)	1.000
Fetal growth restriction Yes No	21 (6.3%) 313 (93.7%)	10 (7.8%) 119 (92.2%)	11 (5.4%) 194 (94.6%)	0.382
Previous preterm birth Yes No	12 (3.6%) 317 (96.4%)	5 (3.9%) 123 (96.1%)	7 (3.4%) 194 (96.6%)	0.842
Previous birth by cesarean section Yes No	150 (44.9%) 181 (55.1%)	53 (41.1%) 75 (58.9%)	97 (47.3%) 106 (52.7%)	0.256
Arterial hypertension Yes No	56 (16.8%) 278 (83.2%)	31 (24.0%) 98 (76.0%)	25 (12.2%) 180 (87.8%)	0.005
Diabetes Yes No	39 (11.7%) 295 (88.3%)	15 (11.6%) 114 (88.4%)	24 (11.7%) 181 (88.3%)	1.000

Abbreviation: ACS, antenatal corticosteroids.

* Value represents χ ^2^ statistic or Fisher test of comparison among two groups for categorical variables

* P-value represents χ ^2^ or Fisher test statistics of comparison among two groups for categorical variables

Considering morbidity at birth, 11.1% of the newborns needed some support of transition at birth, which included oxygen supplementation in 9.6% of the cases, IPPV in 8.4% of the cases, and endotracheal intubation in 1.5% of the cases. There were no differences between the groups regarding all included outcomes related to morbidity at birth ( Table 4 ). Even when analyzed as composite variables – composite morbidity at birth – the differences remained not significant ( Table 4 ).

**Table 4:** Occurrence of primary outcomes in all newborns and comparison between the two groups of interest

*Primary Outcomes*				
Variables	All ( *n * = 334)	Group with ACS ( *n* = 129)	Group without ACS ( *n* = 205)	*p-value* *
**Morbidity at birth**				
Need for oxygen supplementation Yes No	32 (9.6%) 300 (90.4%)	14 (10.8%) 115 (89.2%)	18 (8.9%) 185 (91.1%)	0.961
Need for IPPV Yes No	28 (8.4%) 304 (91.6%)	11 (8.5%) 118 (91.5%)	17 (8.3%) 186 (91.7%)	0.961
Need for EOT Yes No	5 (1.5%) 327 (98.5%)	1 (0.8%) 128 (99.2%)	4 (1.9%) 199 (98.1%)	0.652
Apgar score at the 1 ^st^ minute of life 7–10 0–6	319 (95.8%) 14 (4.2%)	123 (96.9%) 4 (3.1%)	194 (95.1%) 10 (4.9%)	0.425
Apgar score at the 5 ^th^ minute of life 7–10 0–6	330 (99.1%) 3 (0.9%)	127 (98.4%) 2 (1.6%)	203 (99.5%) 1 (0.5%)	0.562
Composite morbidity at birth outcome # Yes No	37 (11.1%) 295 (88.9%)	15 (11.6%) 114 (88.4%)	22 (10.8%) 181 (89.2%)	0.823
**Respiratory morbidity in the first 72 hours**				
Need of oxygen supplementation Yes No	10 (3%) 324 (97%)	5 (3.8%) 124 (96.2%)	5 (2.4%) 200 (97.5%)	0.453
Need for noninvasive respiratory support Yes No	0 (0%) 334 (100%)	0 (0%) 129 (100%)	0 (0%) 205 (100%)	−
Need for invasive respiratory support Yes No	1 (0.3%) 333 (99.7%)	0 (0%) 129 (100%)	1 (0.5%) 204 (99.5%)	1.000
Need for sufactant therapy Yes No	0 (0%) 332 (100%)	0 (0%) 129 (100%)	0 (0%) 203 (100%)	1.000
Newborn RDS Yes No	16 (4.8%) 318 (95.2%)	7 (5.4%) 122 (94.6%)	9 (4.3%) 196 (95.7%)	0.666
TTN Yes No	14 (4.2%) 334 (95.8%)	7 (5.4%) 122 (94.6%)	7 (3.4%) 198 (96.6%)	0.408
Composite respiratory morbidity outcome † Yes No	16 (4.8%) 316 (95.3%)	7 (5.4%) 122 (94.6%)	9 (4.4%) 194 (95.6%)	0.681

Abbreviations: ACS, antenatal corticosteroids; IPPV, intermittent positive airway pressure; RDS, respiratory distress syndrome; TTN, transient tachypnea of the neonate,

* P-value represents χ ^2^ or Fisher test statistics of comparison among two groups for categorical variables.

# Composite morbidity at birth outcome combines oxygen supplementation, need for intermittent positive airway pressure, need for EOT, Apgar score at the 1 ^st^ minute of life < 7 and Apgar score at the 5 ^th^ minute of < 7.

† Composite respiratory morbidity combines need of oxygen supplementation, need of noninvasive respiratory support, need of invasive respiratory support, need of surfactant therapy and newborn respiratory distress syndrome.

Regarding respiratory morbidity in the first 72 hours of life, we observed that 3% of all newborns needed oxygen supplementation, 0.3% needed respiratory ventilation support, while 4.8 and 4.2% presented with RDS and TTN, respectively. None of the newborns needed surfactant therapy (see Table 4 ). When comparing between the two groups, there was no statistically significant difference for these outcomes, even when combined in the composite respiratory morbidity outcome ( Table 4 ).

No statistically significant difference was observed regarding the occurrence of the secondary outcomes in the two study groups ( Table 5 ).

**Table 5:** Occurrence of secondary outcomes in all newborns and comparison between the two groups of interest

*Secondary Outcomes*				
Variables	All ( *n* = 340)	Group with ACS ( *n * = 131)	Group without ACS ( *n * = 209)	*p-value* *
Prolonged hospital staying (in days) 0–5 > 5	329 (98.5%) 5 (1.5%)	128 (99.2%) 1 (0.8%)	201 (98%) 4 (2%)	0.652
Admission at the NIUC				0.436
Yes	7 (2.1%)	4 (3.1%)	3 (1.5%)	
No	328 (97.9%)	126 (96.9%)	202 (98.6%)	
Nutrition through nasogastric tube				1.000
Yes	5 (1.5%)	2 (1.6%)	3 (1.5%)	
No	329 (98.5%)	127 (98.4%)	202 (98.5%)	
Jaundice with need for phototherapy Yes No	15 (4.5%) 319 (95.5%)	8 (6.2%) 121 (93.8%)	7 (3.4%) 198 (96.6%)	0.231
Sepsis evaluation Yes No	22 (6.6%) 312 (93.4%)	6 (4.7%) 123 (95.3%)	16 (7.8%) 189 (92.2%)	0.258
Treatment with antibiotics Yes No	7 (2.1%) 327 (97.9%)	2 (1.6%) 127 (98.4%)	5 (2.4%) 200 (97.6%)	0.711
Hypoglycemia Yes No	10 (3%) 324 (97%)	4 (3.1%) 125 (96.9%)	6 (2.9%) 199 (97.1%)	0.928
General neonatal morbidity outcome # Yes No	38 (11.4%) 296 (88.6%)	14 (10.9%) 115 (89.1%)	24 (11.7%) 181 (88.3%)	0.811

Abbreviation: NICU, neonatal intensive care unit.

* P-value represents χ2 or Fisher test statistics of comparison among two groups for categorical variables.

# General neonatal morbidity outcome combines prolonged hospital staying (in days), admission at the neonatal care unit, nutrition through nasogastric tube, jaundice with need for phototherapy, sepsis evaluation, treatment with antibiotics, and hypoglycemia

When comparing the likelihood for the occurrence of composite morbidity at birth outcome, newborn RDS, TTN, composite respiratory morbidity outcome, NICU admission, prolonged hospital stay (≥ 5 extra days), and composite general neonatal morbidity, the present study did not reveal a statistically significant difference even when adjusting for gestational age and arterial hypertension ( Table 6 ).

**Table 6:** Risk of composite outcomes in neonates with antenatal corticotherapy after adjusting for gestational age and arterial hypertension

Variables	Crude odds ratio (95%CI)	Adjusted odds ratio (95%CI)
**Morbidity at birth**		
Composite morbidity at birth outcome #	1.083 (0.539–2.173)	0.901 (0.422–1.924)
**Respiratory morbidity in the first 72 hours of life**		
Newborn RDS	1.250 (0.454–3.442)	0.732 (0.240–2.232)
TTN	1.623 (0.556–4.739)	0.959 (0.297–3.091)
Composite respiratory morbidity outcome †	1.237 (0.449–3.407)	0.727 (0.239–2.216)
**General neonatal morbidity**		
NICU admission	2.155 (0.474–9.788)	0.852 (0.161–4.520)
Prolonged hospital stay (≥ extra days)	0.393 (0.043–3.552)	0.128 (0.012–1.407)
Composite general neonatal morbidity outcome*	0.918 (0.456–1.848)	0.609 (0.282–1.319)

Abbreviations: CI, confidence interval; NICU, neonatal intensive care unit; RDS, respiratory distress syndrome; TTN, transient tachypnea of the neonate,

# Composite morbidity at birth outcome combines oxygen supplementation, , need for intermittent positive airway pressure , need for EOT, Apgar score at the 1 ^st^ minute of life < 7 and Apgar score at the 5 ^th^ minute of life < 7.

† Composite respiratory morbidity combines need of oxygen supplementation, need of noninvasive respiratory support, need of invasive respiratory support, need of sufactant therapy, newborn respiratory distress syndrome, and transient tachypnea of the neonate.

* General neonatal morbidity outcome combines prolonged hospital stay (in days), admission at the neonatal care unit, nutrition through nasogastric tube, jaundice with need for phototherapy, sepsis evaluation, treatment with antibiotics, and hypoglycemia

## Discussion

There is not much available data regarding the rates of elective CS. It is believed that, due to the management of previous CS and breech presentation, elective CS has been increasing. Some studies report rates of elective CS of between 7.4 and 13%. 4 5 The population of the study represents 4.6% of all neonates born in the studied period, and the main indication for elective CS ( Table 1 ) was previous uterine surgery, essentially previous CS, which is similar to what was observed in the randomized cohort studies assessing ACS before elective term CS.

Regarding obstetrical risk factors, our study revealed differences between the ACS-exposed and not exposed group in only 3 of the 12 variables included, namely: short cervix, threatened preterm birth, and arterial hypertension. We believe that some of these differences may represent confounding by indication that is, we believe that mothers received ACS based on their underlying risk profile.

Regarding the outcomes regarding morbidity at birth, the overall incidence observed in the present study was very similar to those reported in the randomized cohort studies assessing ACS before an elective CS. 19 20 21 22 Nada et al. 20 and Nooh et al. 21 presented a slightly higher overall incidence of AS inferior to seven at the first and fifth minutes, but they also did not find a statistical significant difference between the ACS exposed and not exposed group. Stutchfield et al. 22 reported a lower overall rate of IPPV and endotracheal intubation, but they also did not observe a statistical significant difference between de ACS exposed and not exposed group. Regarding the occurrence of RDS this study also revealed similar rates to those described in the literature, 19 20 21 22 and similar to Nada et al. 20 and Nooh et al., 21 we also did not observe a statistically significant difference between the ACS-exposed and not exposed groups. In our study, there was no statistically significant difference between the groups regarding the incidence of TTN, as opposed to what was observed in the randomized cohort studies available in the literature, where the incidence of TTN was lower in the ACS exposed group, with a statistically significant difference. 19 20 21 22 The studies available also reported, contrary to us, higher rates of NICU admission and prolonged hospital stay in the ACS not exposed group, although with conflicting results regarding statistical relevance. 19 20 21 22 None of the randomized cohort studies reported data regarding nutrition through nasogastric tube, jaundice with need for phototherapy, sepsis evaluation, treatment with antibiotics, or hypoglycemia. 19 20 21 22 23 Our findings regarding higher rates of hypoglycemia in the ACS-exposed group may reflect the known biological effects of steroids in glycemic profiles.

Finally, according to the results of the aforementioned randomized cohort studies, prophylactic ACS appeared to decrease the risk of RDS (risk ratio [RR] = 0.48; 95%CI = 0.27–0.87; 3,817 participants), TTN (RR = 0.43; 95%CI: 0.29–0.65; 3,821 participants), and admission to NICU for morbidity due to respiratory reasons (RR = 0.42; 95%CI: 0.22–0.79; 3 studies; 3,441 participants), or any indication (RR = 0.14; 95%CI: 0.03–0.61; 1 study; 452 participants), and the length of stay in the NICU by 2.70 days (mean difference [MD] - 2.70; 95%CI: - 2.76–-2.64; 2 studies; 32 participants). 19 20 21 22 23 The present study found no statistically significant difference between the exposed and not exposed group regarding the likelihood of newborn RDS, TTN, and NICU admission, and even when adjusting for gestational age and arterial hypertension, the likelihood for the occurrence of the aforementioned outcomes was not statistically different between the exposed and not exposed newborns.

Besides confounding by indication, some other limitations can partially explain the results encountered. One is the suboptimal exposure to ACS. It is known that the optimal window to birth is between 2 and 7 days after the ACS cycle. 13 Nonetheless, even when ACS is formally indicated, evidence shows that < 40% of the women delivered in this time window. 24 25 In our study, although the ACS cycle was completed in 93.7% of the exposed newborns, 62.4% ( *n* = 65) of these fell outside the time window in which ACS are most effective. Finally, the retrospective nature of the present study warrants a careful interpretation of the results.

## Conclusion

In conclusion, in elective CS between 37 and 38 + 6 weeks of gestation, there seems to be no differences regarding newborn outcomes between those exposed and not exposed to ACS. Therefore, the findings of the present study cannot support the use of ACS in this group. More studies regarding this topic, specifically prospective ones, are needed to validate this conclusion.
